# Complete Genome Sequences of Mycoplasma ovipneumoniae Strains 150 and 274, Isolated from Different Regions in Bosnia and Herzegovina

**DOI:** 10.1128/mra.00011-23

**Published:** 2023-02-27

**Authors:** Huafang Hao, Zinka Maksimović, Lina Ma, Maid Rifatbegović, Shengli Chen, Xinmin Yan, Lei Fu, Yuefeng Chu

**Affiliations:** a State Key Laboratory of Veterinary Etiological Biology, College of Veterinary Medicine, Lanzhou University, Lanzhou Veterinary Research Institute, Chinese Academy of Agricultural Sciences, Lanzhou, People’s Republic of China; b Department of Pathobiology and Epidemiology, Veterinary Faculty, University of Sarajevo, Sarajevo, Bosnia and Herzegovina; University of Maryland School of Medicine

## Abstract

Mycoplasma ovipneumoniae is an important pathogen in sheep, goats, and wild ruminants. We sequenced *M. ovipneumoniae* strains 150 and 274 from Bosnia and Herzegovina. Strain 150 has a circular genome of 1,053,380 bp with 29.15% GC content while strain 274 has 1,081,404 bp with 28.82% GC content.

## ANNOUNCEMENT

Mycoplasma ovipneumoniae is a primary pathogenic agent of respiratory tracts that may cause lethal pneumonia in sheep and goats (1–4), which has been reported as the most frequently isolated species in sheep and lambs with pneumonia (3, 5–8). *M. ovipneumoniae* infection and prevalence have led to large financial losses worldwide in the sheep industry (9–11).

*M. ovipneumoniae* strain 150 was isolated from a nasal swab of a sheep with respiratory disease in Central Bosnia Canton (Bosnia and Herzegovina [B&H]) in 2009, and strain 274 was isolated from the lung of an apparently healthy goat in Herzegovina-Neretva Canton (B&H) in 2010 (12, 13). Both were cultured in Thiaucourt’s liquid and solid medium (14) and incubated in a 95% N_2_ and 5% CO_2_ humidified atmosphere at 37°C until growth, and the isolates were identified by PCR of 16S rRNA gene without sequencing (5). They were inoculated into Thiaucourt’s medium and grown at 37°C for 72 h, and genomic DNA was extracted using the DNeasy Blood & Tissue kit (Qiagen, Valencia, CA). The DNA samples were detected by agarose gel electrophoresis and then sheared in a Covaris g-Tube to obtain 15-kb fragments. DNA was used to prepare a barcoded SMRTbell library with the SMRTbell Express template preparation kit v2.0, the libraries were quantified by Qubit, and the insertion size was detected using Bioanalyzer 2100, and DNA was sequenced with the PacBio Sequel II system at Berry Genomics (Beijing, China) with default parameters. The raw reads were processed for quality to remove adaptor sequences and filter out low-quality and short-length reads using SMRT Link v10.1 analysis with default parameters (15). Genome assembly was performed using Canu v1.6 (16) and polished by GCpp v2.0.0 (17). MUMmer v4.0 (18) was used for self-alignment, and the genome was circularized by a collinear relationship between head and tail sequence with default parameters. Protein-coding genes were predicted with Prodigal v2.63 (-g was set as “4”) (19). tRNAs, rRNA, and small RNAs (sRNAs) were predicted by tRNAscan-SE v1.3.1 (20), BLAST v2.2.25 against homologous sequences, and the Rfam v12.1 database (21), respectively. Default parameters were used for all software unless otherwise specified.

A total of 177,992 reads totaling 2,350,445,533 bases were obtained, resulting in 2,350× genome coverage for strain 150, and *N*_50_ read length was 13,975 bp. For strain 274, 168,529 reads totaling 2,150,600,373 bases were obtained, resulting in 2,150× genome coverage, and *N*_50_ read length was 13,510 bp. The whole-genome sequences of 150 and 274 are 1,053,380 bp and 1,081,404 bp separately in length with GC content of 29.15% and 28.82%, respectively. Both genomes were individually composed of a single circular chromosome. Strain 150 has 714 protein-coding genes, 30 tRNAs, 3 rRNAs, and 2 sRNAs while strain 274 contains 747 protein-coding genes, 30 tRNAs, 3 rRNAs, and 2 sRNAs.

### Data availability.

The genome sequences of *M. ovipneumoniae* strains 274 and 150 were deposited in GenBank under the accession numbers CP079199 and CP079200, respectively. The respective raw reads can be found under the accession numbers SRR22071529 and SRR22071683.
